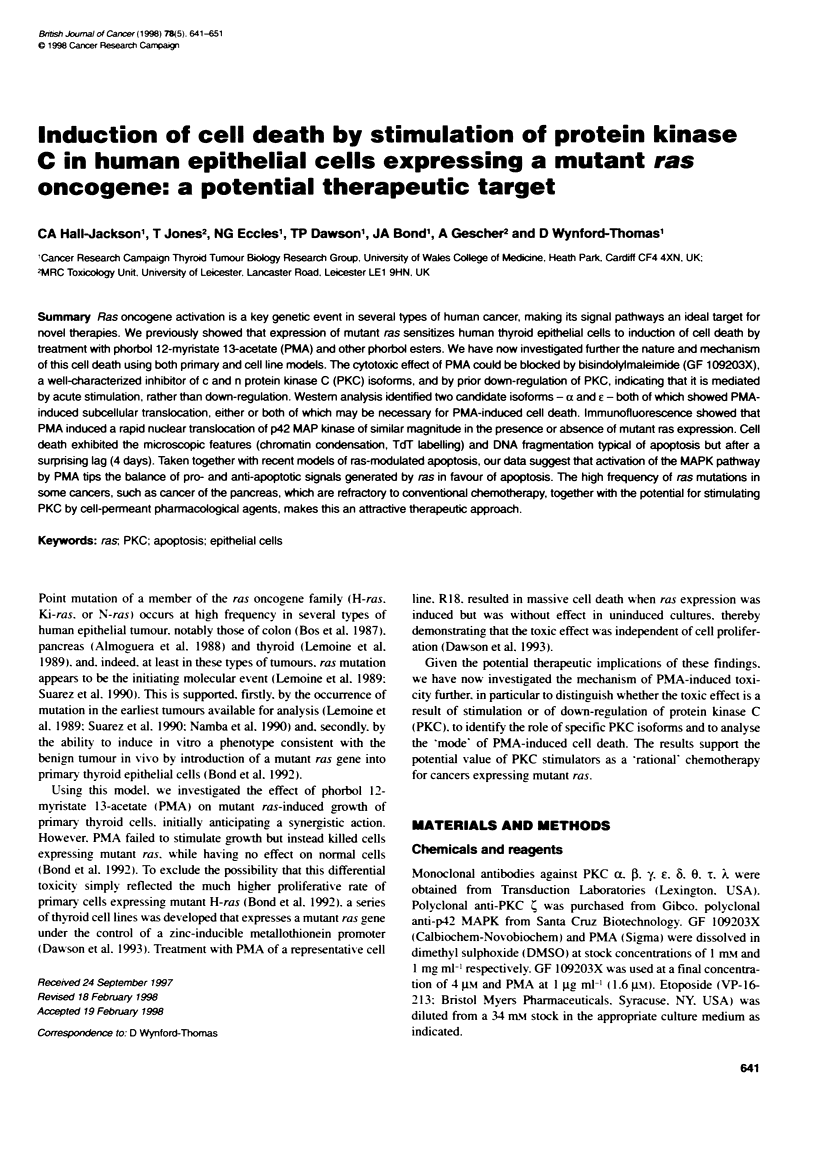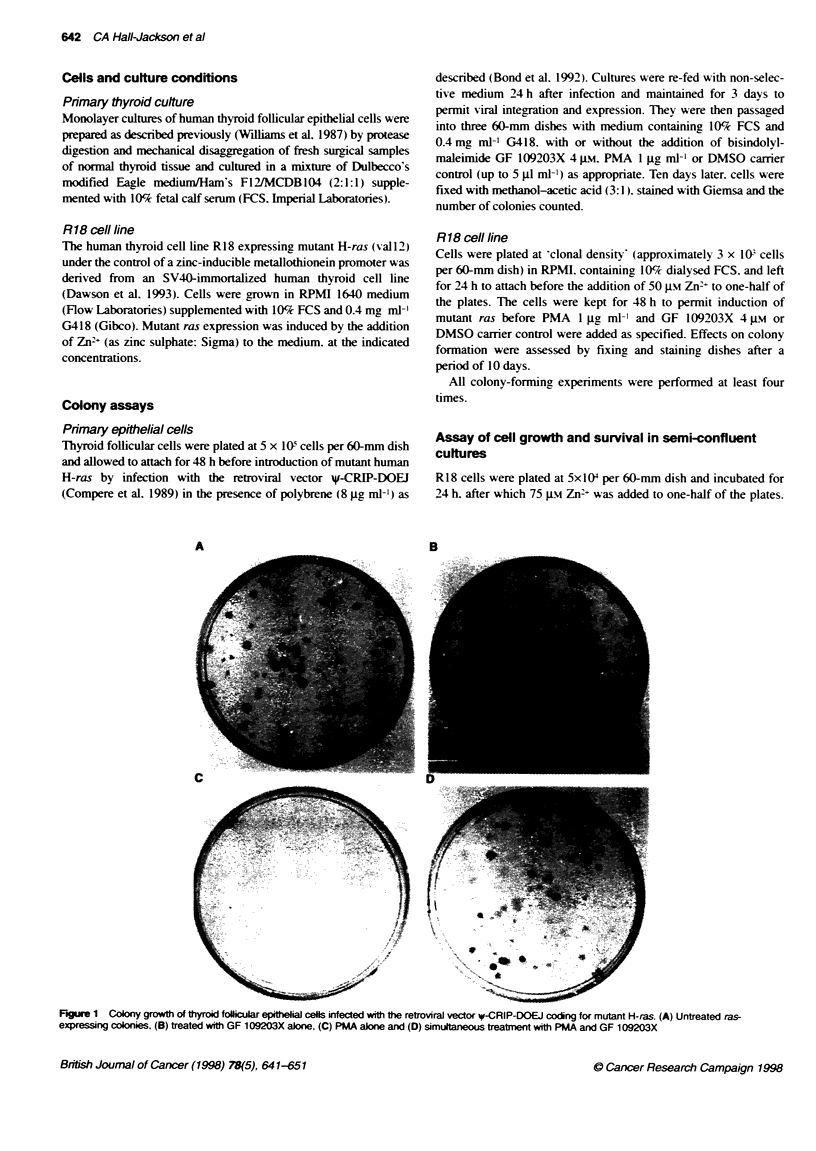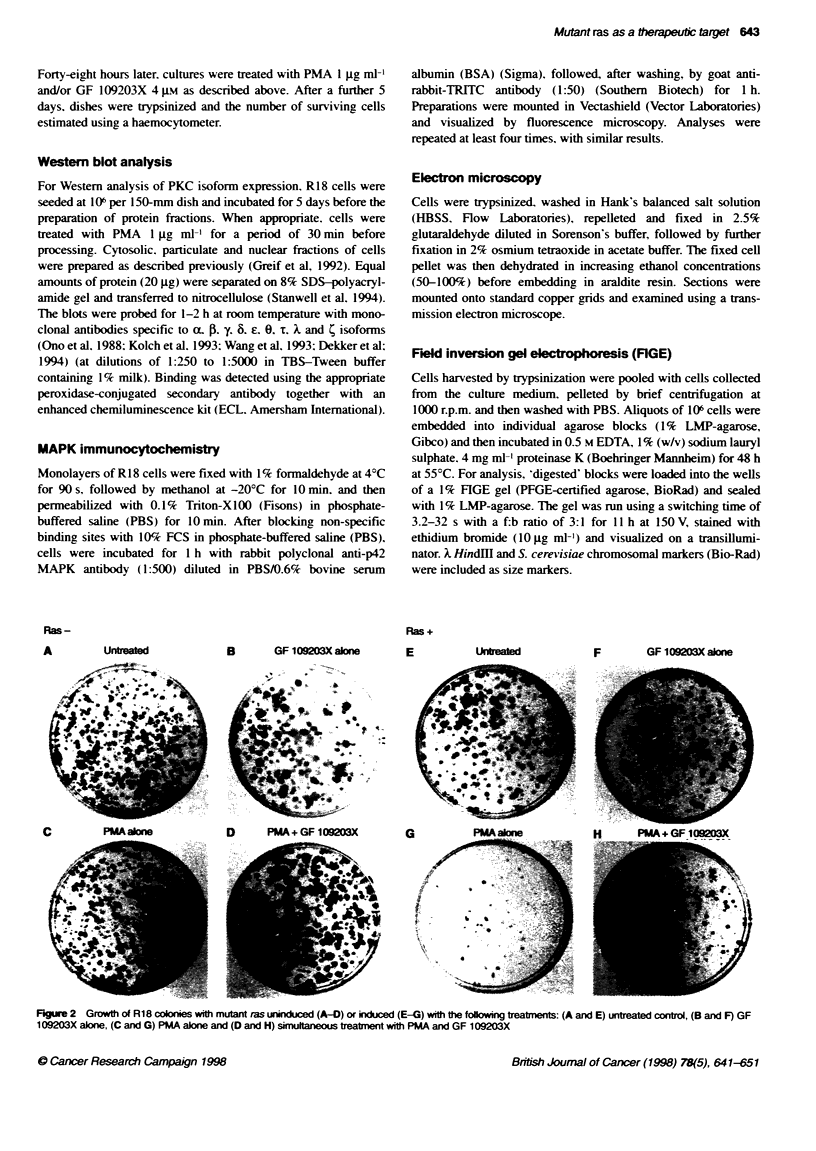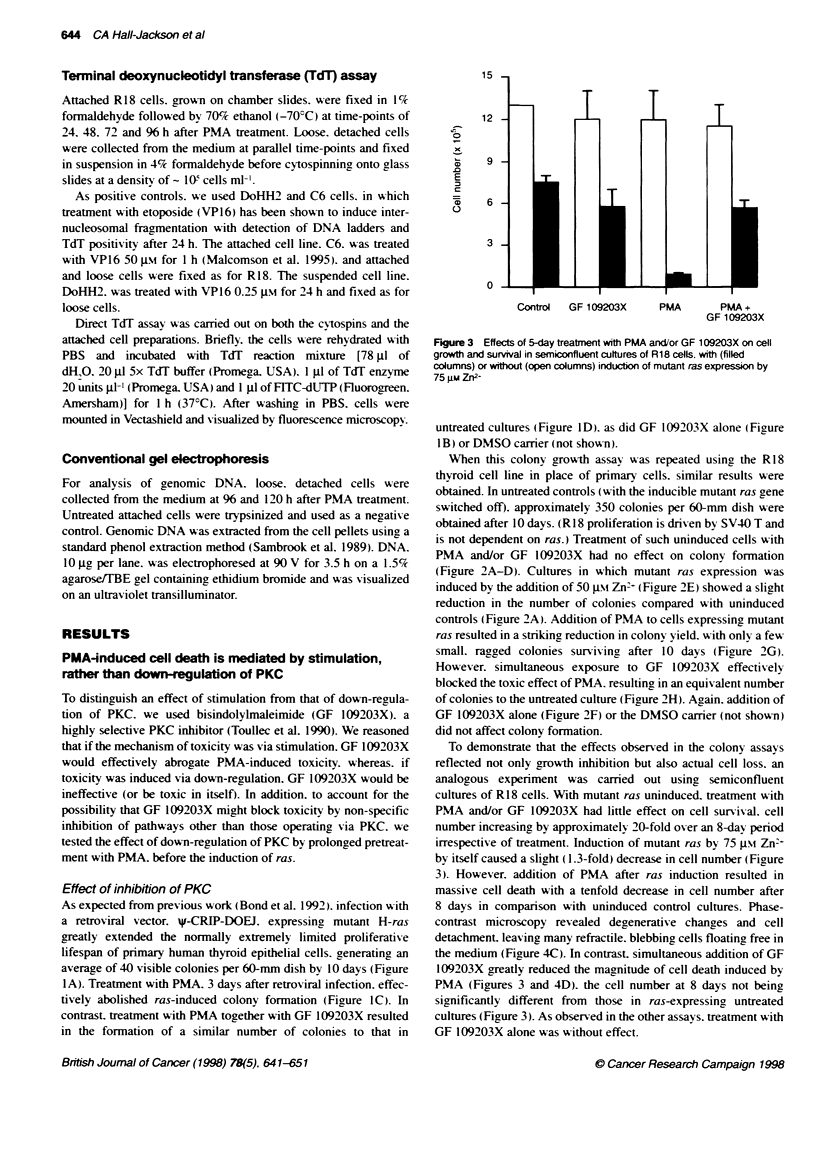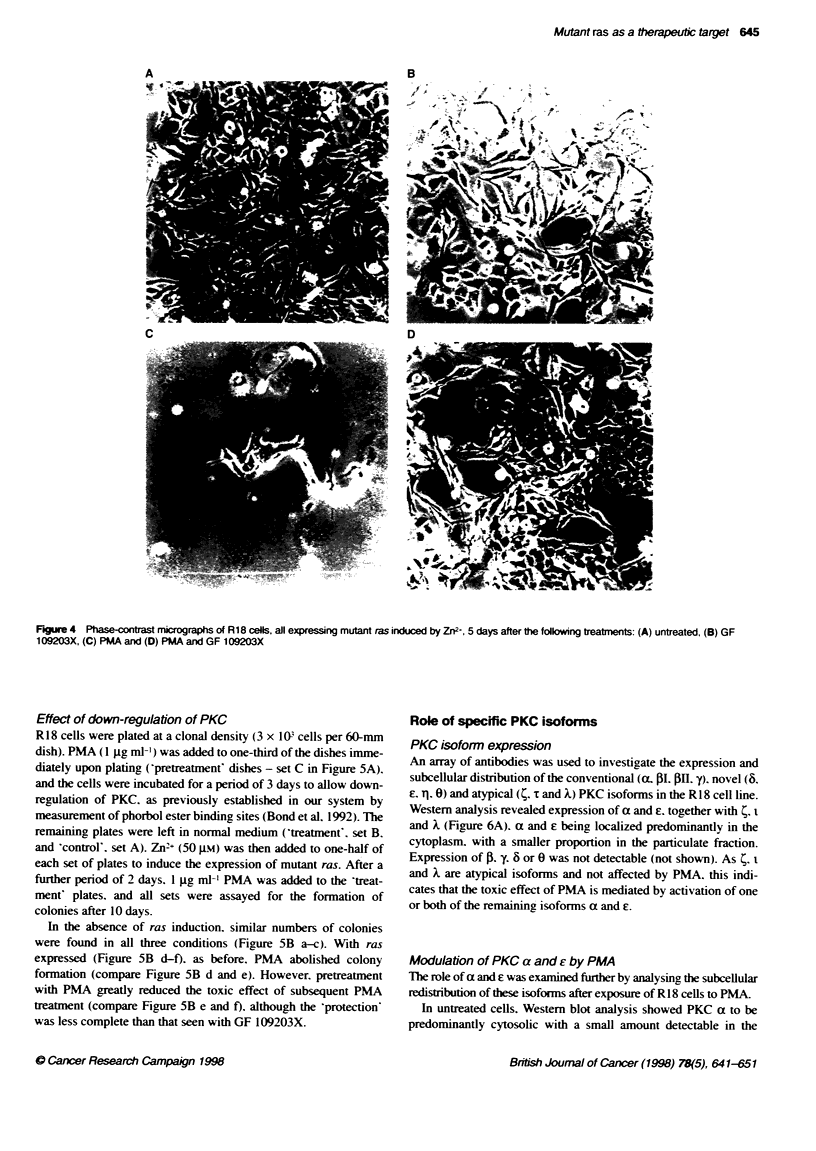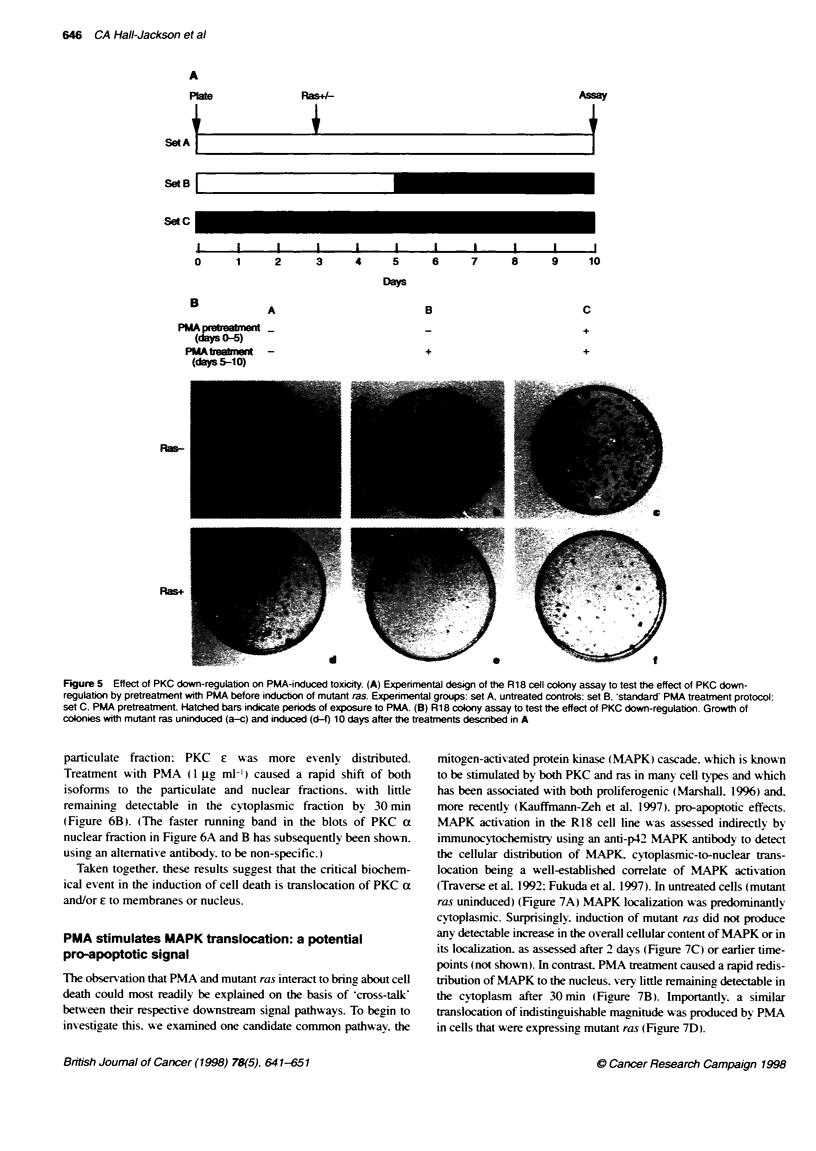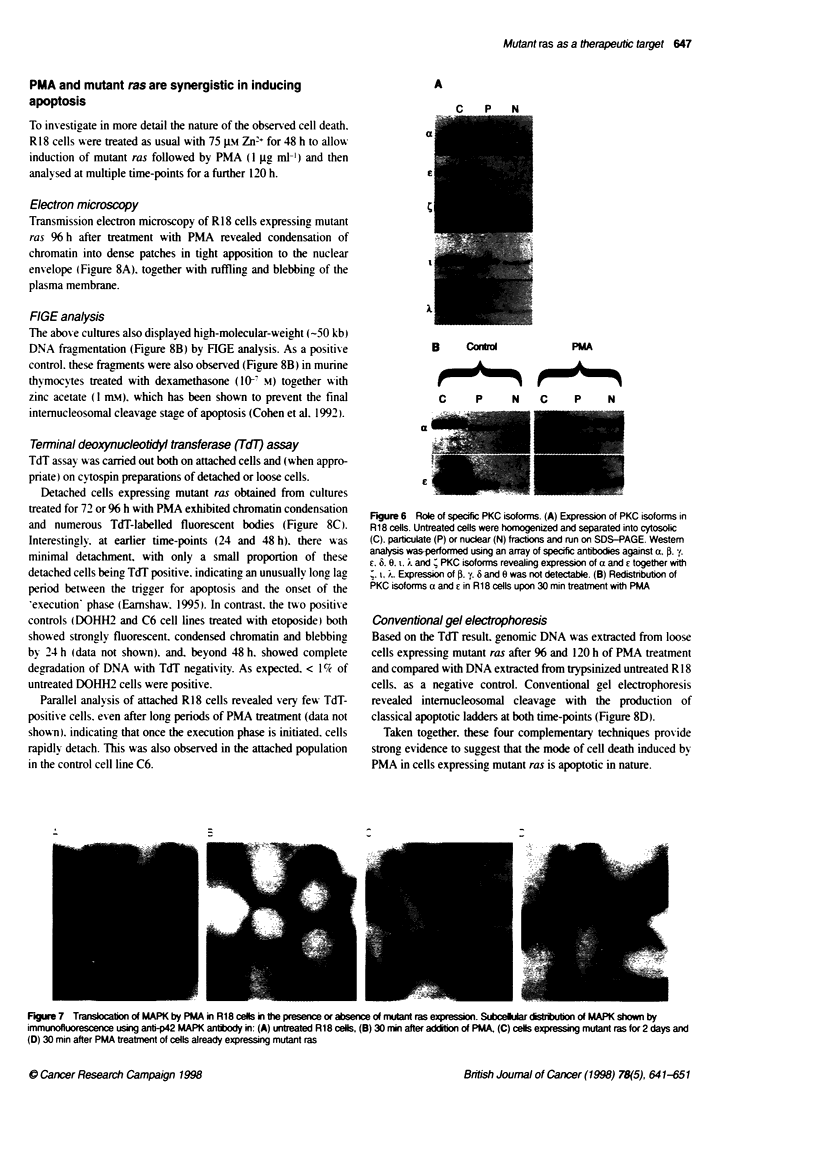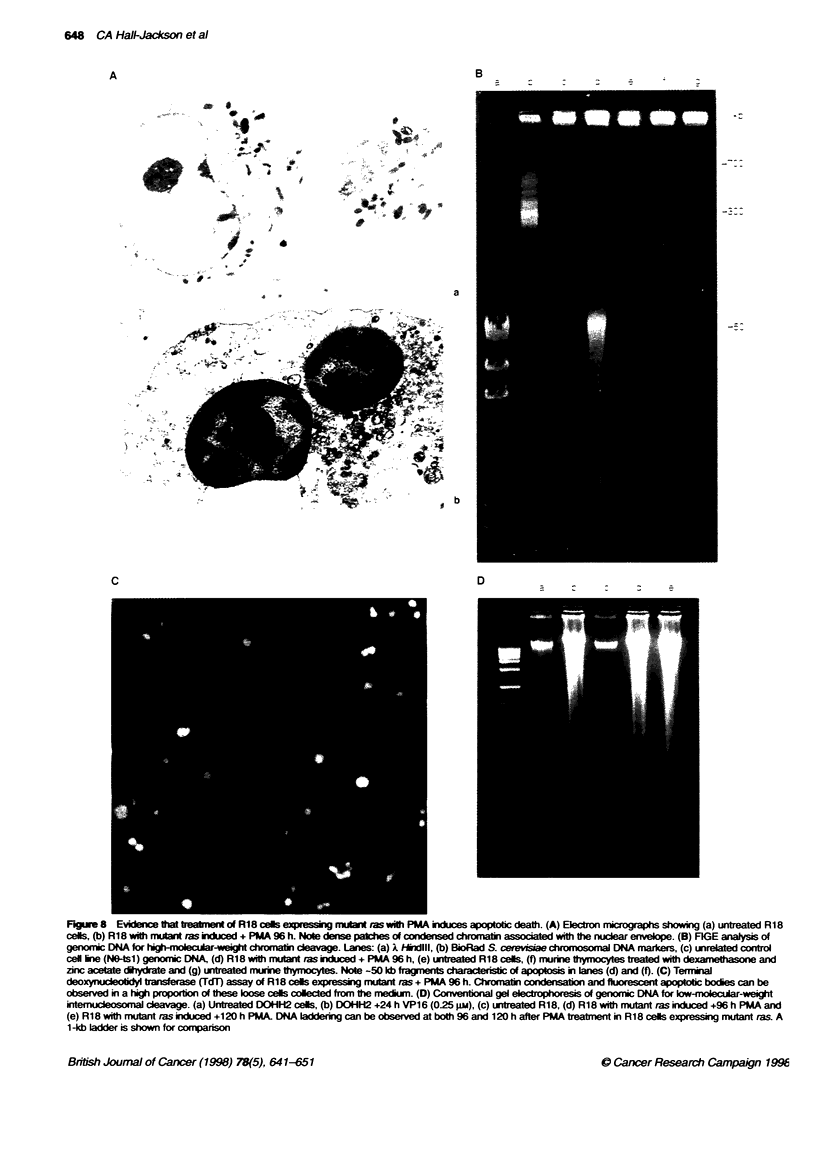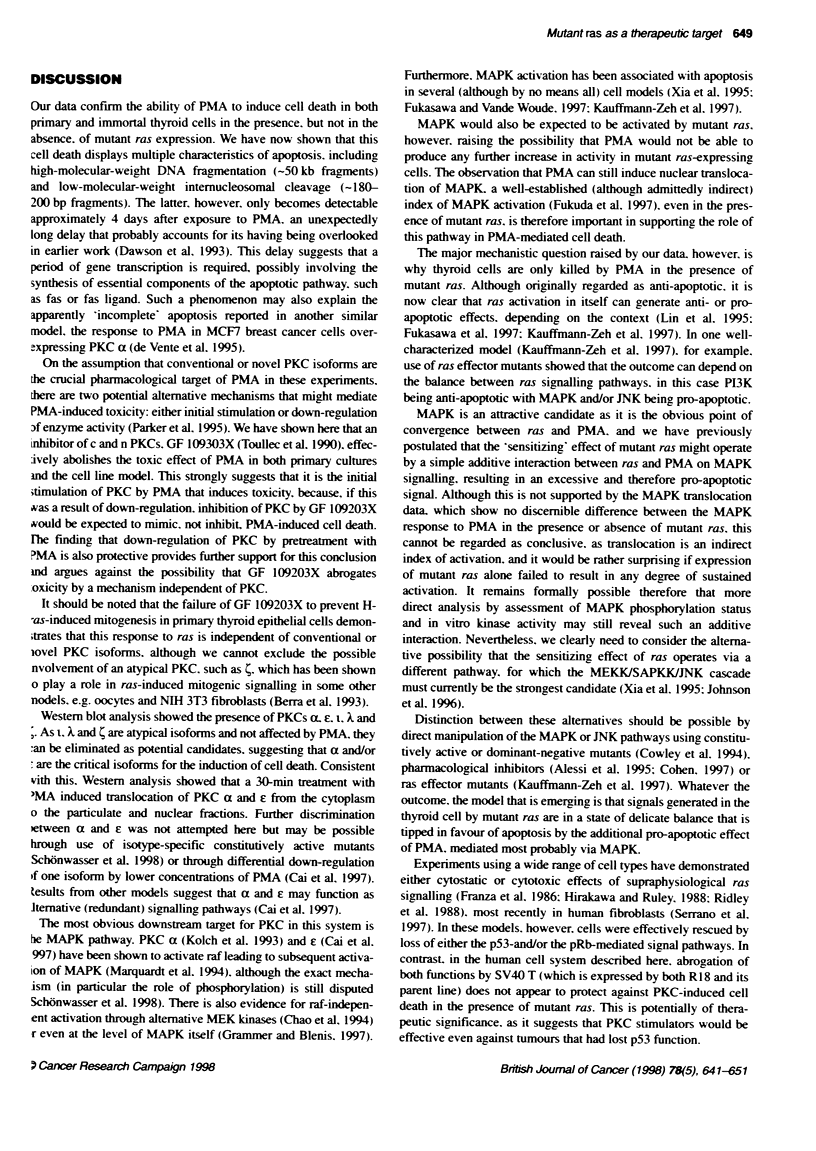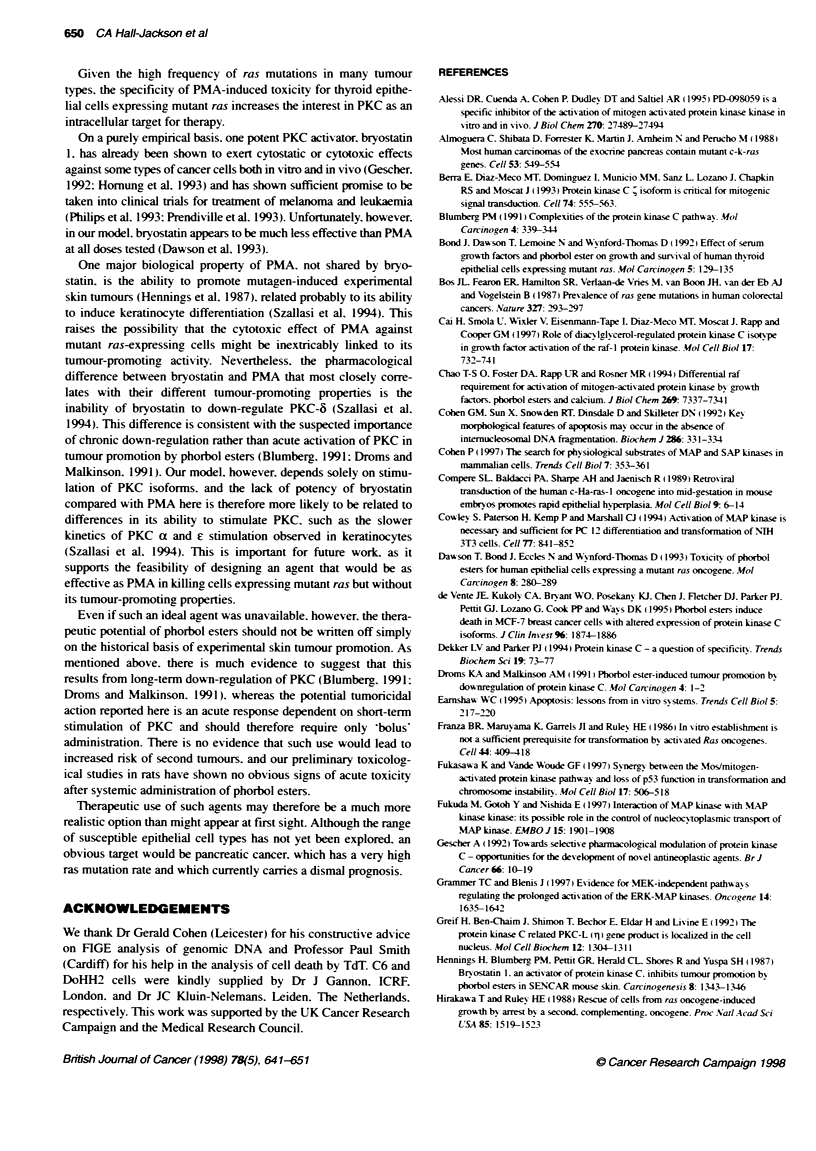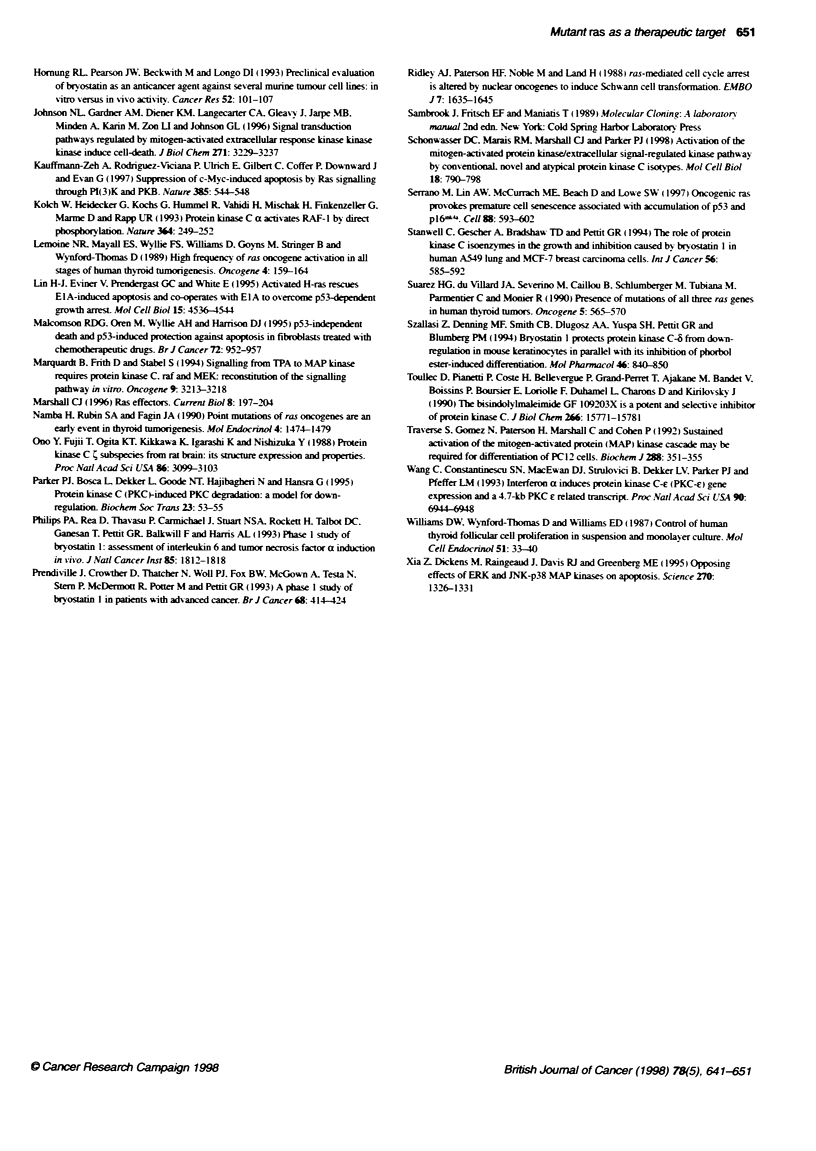# Induction of cell death by stimulation of protein kinase C in human epithelial cells expressing a mutant ras oncogene: a potential therapeutic target.

**DOI:** 10.1038/bjc.1998.554

**Published:** 1998-09

**Authors:** C. A. Hall-Jackson, T. Jones, N. G. Eccles, T. P. Dawson, J. A. Bond, A. Gescher, D. Wynford-Thomas

**Affiliations:** Cancer Research Campaign Thyroid Tumour Biology Research Group, University of Wales College of Medicine, Cardiff, UK.

## Abstract

**Images:**


British Journal of Cancer (1998) 78(5), 641–651
© 1998 Cancer Research Campaign

### Induction of cell death by stimulation of protein kinase C in human epithelial cells expressing a mutant *ras* oncogene: a potential therapeutic target

**CA Hall-Jackson[1], T Jones[2], NG Eccles[1], TP Dawson[1], JA Bond[1], A Gescher[2] and D Wynford-Thomas[1]**

[1]Cancer Research Campaign Thyroid Tumour Biology Research Group, University of Wales College of Medicine, Heath Park, Cardiff CF4 4XN, UK;
[2]MRC Toxicology Unit, University of Leicester, Lancaster Road, Leicester LE1 9HN, UK

**Summary** *Ras* oncogene activation is a key genetic event in several types of human cancer, making its signal pathways an ideal target for novel therapies. We previously showed that expression of mutant *ras* sensitizes human thyroid epithelial cells to induction of cell death by treatment with phorbol 12-myristate 13-acetate (PMA) and other phorbol esters. We have now investigated further the nature and mechanism of this cell death using both primary and cell line models. The cytotoxic effect of PMA could be blocked by bisindolylmaleimide (GF 109203X), a well-characterized inhibitor of c and n protein kinase C (PKC) isoforms, and by prior down-regulation of PKC, indicating that it is mediated by acute stimulation, rather than down-regulation. Western analysis identified two candidate isoforms – α and ε – both of which showed PMA-induced subcellular translocation, either or both of which may be necessary for PMA-induced cell death. Immunofluorescence showed that PMA induced a rapid nuclear translocation of p42 MAP kinase of similar magnitude in the presence or absence of mutant ras expression. Cell death exhibited the microscopic features (chromatin condensation, TdT labelling) and DNA fragmentation typical of apoptosis but after a surprising lag (4 days). Taken together with recent models of ras-modulated apoptosis, our data suggest that activation of the MAPK pathway by PMA tips the balance of pro- and anti-apoptotic signals generated by *ras* in favour of apoptosis. The high frequency of *ras* mutations in some cancers, such as cancer of the pancreas, which are refractory to conventional chemotherapy, together with the potential for stimulating PKC by cell-permeant pharmacological agents, makes this an attractive therapeutic approach.

**Keywords:** *ras*; PKC; apoptosis; epithelial cells

Point mutation of a member of the *ras* oncogene family (H-*ras*, Ki-*ras*, or N-*ras*) occurs at high frequency in several types of human epithelial tumour, notably those of colon (Bos et al, 1987), pancreas (Almoguera et al, 1988) and thyroid (Lemoine et al, 1989), and, indeed, at least in these types of tumours, *ras* mutation appears to be the initiating molecular event (Lemoine et al, 1989; Suarez et al, 1990). This is supported, firstly, by the occurrence of mutation in the earliest tumours available for analysis (Lemoine et al, 1989; Suarez et al, 1990; Namba et al, 1990) and, secondly, by the ability to induce in vitro a phenotype consistent with the benign tumour in vivo by introduction of a mutant *ras* gene into primary thyroid epithelial cells (Bond et al, 1992).

Using this model, we investigated the effect of phorbol 12-myristate 13-acetate (PMA) on mutant *ras*-induced growth of primary thyroid cells, initially anticipating a synergistic action. However, PMA failed to stimulate growth but instead killed cells expressing mutant *ras*, while having no effect on normal cells (Bond et al, 1992). To exclude the possibility that this differential toxicity simply reflected the much higher proliferative rate of primary cells expressing mutant H-*ras* (Bond et al, 1992), a series of thyroid cell lines was developed that expresses a mutant *ras* gene under the control of a zinc-inducible metallothionein promoter (Dawson et al, 1993). Treatment with PMA of a representative cell line, R18, resulted in massive cell death when *ras* expression was induced but was without effect in uninduced cultures, thereby demonstrating that the toxic effect was independent of cell proliferation (Dawson et al, 1993).

Given the potential therapeutic implications of these findings, we have now investigated the mechanism of PMA-induced toxicity further, in particular to distinguish whether the toxic effect is a result of stimulation or of down-regulation of protein kinase C (PKC), to identify the role of specific PKC isoforms and to analyse the 'mode' of PMA-induced cell death. The results support the potential value of PKC stimulators as a 'rational' chemotherapy for cancers expressing mutant *ras*.

*Received 24 September 1997*
*Revised 18 February 1998*
*Accepted 19 February 1998*

*Correspondence to:* D Wynford-Thomas

### MATERIALS AND METHODS

### Chemicals and reagents

Monoclonal antibodies against PKC α, β, γ, ε, δ, θ, τ, λ were obtained from Transduction Laboratories (Lexington, USA). Polyclonal anti-PKC ζ was purchased from Gibco, polyclonal anti-p42 MAPK from Santa Cruz Biotechnology. GF 109203X (Calbiochem-Novobiochem) and PMA (Sigma) were dissolved in dimethyl sulphoxide (DMSO) at stock concentrations of 1 mM and 1 mg ml$^{-1}$ respectively. GF 109203X was used at a final concentration of 4 μM and PMA at 1 μg ml$^{-1}$ (1.6 μM). Etoposide (VP-16-213; Bristol Myers Pharmaceuticals, Syracuse, NY, USA) was diluted from a 34 mM stock in the appropriate culture medium as indicated.

### Cells and culture conditions

### Primary thyroid culture

Monolayer cultures of human thyroid follicular epithelial cells were prepared as described previously (Williams et al, 1987) by protease digestion and mechanical disaggregation of fresh surgical samples of normal thyroid tissue and cultured in a mixture of Dulbecco's modified Eagle medium/Ham's F12/MCDB104 (2:1:1) supplemented with 10% fetal calf serum (FCS, Imperial Laboratories).

### R18 cell line

The human thyroid cell line R18 expressing mutant H-*ras* (val12) under the control of a zinc-inducible metallothionein promoter was derived from an SV40-immortalized human thyroid cell line (Dawson et al, 1993). Cells were grown in RPMI 1640 medium (Flow Laboratories) supplemented with 10% FCS and 0.4 mg $ml^{-1}$ G418 (Gibco). Mutant *ras* expression was induced by the addition of $Zn^{2+}$ (as zinc sulphate: Sigma) to the medium, at the indicated concentrations.

### Colony assays

### Primary epithelial cells

Thyroid follicular cells were plated at $5 \times 10^5$ cells per 60-mm dish and allowed to attach for 48 h before introduction of mutant human H-*ras* by infection with the retroviral vector ψ-CRIP-DOEJ (Compere et al, 1989) in the presence of polybrene (8 μg $ml^{-1}$) as described (Bond et al, 1992). Cultures were re-fed with non-selective medium 24 h after infection and maintained for 3 days to permit viral integration and expression. They were then passaged into three 60-mm dishes with medium containing 10% FCS and 0.4 mg $ml^{-1}$ G418, with or without the addition of bisindolylmaleimide GF 109203X 4 μM, PMA 1 μg $ml^{-1}$ or DMSO carrier control (up to 5 μl $ml^{-1}$) as appropriate. Ten days later, cells were fixed with methanol–acetic acid (3:1), stained with Giemsa and the number of colonies counted.

### R18 cell line

Cells were plated at 'clonal density' (approximately $3 \times 10^3$ cells per 60-mm dish) in RPMI, containing 10% dialysed FCS, and left for 24 h to attach before the addition of 50 μM $Zn^{2+}$ to one-half of the plates. The cells were kept for 48 h to permit induction of mutant *ras* before PMA 1 μg $ml^{-1}$ and GF 109203X 4 μM or DMSO carrier control were added as specified. Effects on colony formation were assessed by fixing and staining dishes after a period of 10 days.

All colony-forming experiments were performed at least four times.

### Assay of cell growth and survival in semi-confluent cultures

R18 cells were plated at $5 \times 10^4$ per 60-mm dish and incubated for 24 h, after which 75 μM $Zn^{2+}$ was added to one-half of the plates.

**Figure 1** Colony growth of thyroid follicular epithelial cells infected with the retroviral vector ψ-CRIP-DOEJ coding for mutant H-*ras*. (**A**) Untreated *ras*-expressing colonies, (**B**) treated with GF 109203X alone, (**C**) PMA alone and (**D**) simultaneous treatment with PMA and GF 109203X

© Cancer Research Campaign 1998

Forty-eight hours later, cultures were treated with PMA 1 μg $ml^{-1}$ and/or GF 109203X 4 μM as described above. After a further 5 days, dishes were trypsinized and the number of surviving cells estimated using a haemocytometer.

### Western blot analysis

For Western analysis of PKC isoform expression, R18 cells were seeded at $10^6$ per 150-mm dish and incubated for 5 days before the preparation of protein fractions. When appropriate, cells were treated with PMA 1 μg $ml^{-1}$ for a period of 30 min before processing. Cytosolic, particulate and nuclear fractions of cells were prepared as described previously (Greif et al, 1992). Equal amounts of protein (20 μg) were separated on 8% SDS–polyacrylamide gel and transferred to nitrocellulose (Stanwell et al, 1994). The blots were probed for 1–2 h at room temperature with monoclonal antibodies specific to α, β, γ, δ, ε, θ, τ, λ and ζ isoforms (Ono et al, 1988; Kolch et al, 1993; Wang et al, 1993; Dekker et al; 1994) (at dilutions of 1:250 to 1:5000 in TBS–Tween buffer containing 1% milk). Binding was detected using the appropriate peroxidase-conjugated secondary antibody together with an enhanced chemiluminescence kit (ECL, Amersham International).

### MAPK immunocytochemistry

Monolayers of R18 cells were fixed with 1% formaldehyde at 4°C for 90 s, followed by methanol at –20°C for 10 min, and then permeabilized with 0.1% Triton-X100 (Fisons) in phosphate-buffered saline (PBS) for 10 min. After blocking non-specific binding sites with 10% FCS in phosphate-buffered saline (PBS), cells were incubated for 1 h with rabbit polyclonal anti-p42 MAPK antibody (1:500) diluted in PBS/0.6% bovine serum albumin (BSA) (Sigma), followed, after washing, by goat anti-rabbit-TRITC antibody (1:50) (Southern Biotech) for 1 h. Preparations were mounted in Vectashield (Vector Laboratories) and visualized by fluorescence microscopy. Analyses were repeated at least four times, with similar results.

### Electron microscopy

Cells were trypsinized, washed in Hank's balanced salt solution (HBSS, Flow Laboratories), repelleted and fixed in 2.5% glutaraldehyde diluted in Sorenson's buffer, followed by further fixation in 2% osmium tetraoxide in acetate buffer. The fixed cell pellet was then dehydrated in increasing ethanol concentrations (50–100%) before embedding in araldite resin. Sections were mounted onto standard copper grids and examined using a transmission electron microscope.

### Field inversion gel electrophoresis (FIGE)

Cells harvested by trypsinization were pooled with cells collected from the culture medium, pelleted by brief centrifugation at 1000 r.p.m. and then washed with PBS. Aliquots of $10^6$ cells were embedded into individual agarose blocks (1% LMP-agarose, Gibco) and then incubated in 0.5 M EDTA, 1% (w/v) sodium lauryl sulphate, 4 mg $ml^{-1}$ proteinase K (Boehringer Mannheim) for 48 h at 55°C. For analysis, 'digested' blocks were loaded into the wells of a 1% FIGE gel (PFGE-certified agarose, BioRad) and sealed with 1% LMP-agarose. The gel was run using a switching time of 3.2–32 s with a f:b ratio of 3:1 for 11 h at 150 V, stained with ethidium bromide (10 μg $ml^{-1}$) and visualized on a transilluminator. λ *Hind*III and *S. cerevisiae* chromosomal markers (Bio-Rad) were included as size markers.

**Figure 2** Growth of R18 colonies with mutant *ras* uninduced (**A**–**D**) or induced (**E**–**G**) with the following treatments: (**A** and **E**) untreated control, (**B** and **F**) GF 109203X alone, (**C** and **G**) PMA alone and (**D** and **H**) simultaneous treatment with PMA and GF 109203X

© *Cancer Research Campaign 1998*

### Terminal deoxynucleotidyl transferase (TdT) assay

Attached R18 cells, grown on chamber slides, were fixed in 1% formaldehyde followed by 70% ethanol (−70°C) at time-points of 24, 48, 72 and 96 h after PMA treatment. Loose, detached cells were collected from the medium at parallel time-points and fixed in suspension in 4% formaldehyde before cytospinning onto glass slides at a density of ~ $10^5$ cells $ml^{-1}$.

As positive controls, we used DoHH2 and C6 cells, in which treatment with etoposide (VP16) has been shown to induce internucleosomal fragmentation with detection of DNA ladders and TdT positivity after 24 h. The attached cell line, C6, was treated with VP16 50 μM for 1 h (Malcomson et al, 1995), and attached and loose cells were fixed as for R18. The suspended cell line, DoHH2, was treated with VP16 0.25 μM for 24 h and fixed as for loose cells.

Direct TdT assay was carried out on both the cytospins and the attached cell preparations. Briefly, the cells were rehydrated with PBS and incubated with TdT reaction mixture [78 μl of $dH_2O$, 20 μl 5× TdT buffer (Promega, USA), 1 μl of TdT enzyme 20 units $\mu l^{-1}$ (Promega, USA) and 1 μl of FITC-dUTP (Fluorogreen, Amersham)] for 1 h (37°C). After washing in PBS, cells were mounted in Vectashield and visualized by fluorescence microscopy.

### Conventional gel electrophoresis

For analysis of genomic DNA, loose, detached cells were collected from the medium at 96 and 120 h after PMA treatment. Untreated attached cells were trypsinized and used as a negative control. Genomic DNA was extracted from the cell pellets using a standard phenol extraction method (Sambrook et al, 1989). DNA, 10 μg per lane, was electrophoresed at 90 V for 3.5 h on a 1.5% agarose/TBE gel containing ethidium bromide and was visualized on an ultraviolet transilluminator.

### RESULTS

### PMA-induced cell death is mediated by stimulation, rather than down-regulation of PKC

To distinguish an effect of stimulation from that of down-regulation of PKC, we used bisindolylmaleimide (GF 109203X), a highly selective PKC inhibitor (Toullec et al, 1990). We reasoned that if the mechanism of toxicity was via stimulation, GF 109203X would effectively abrogate PMA-induced toxicity, whereas, if toxicity was induced via down-regulation, GF 109203X would be ineffective (or be toxic in itself). In addition, to account for the possibility that GF 109203X might block toxicity by non-specific inhibition of pathways other than those operating via PKC, we tested the effect of down-regulation of PKC by prolonged pretreatment with PMA, before the induction of *ras*.

#### *Effect of inhibition of PKC*

As expected from previous work (Bond et al, 1992), infection with a retroviral vector, ψ-CRIP-DOEJ, expressing mutant H-*ras* greatly extended the normally extremely limited proliferative lifespan of primary human thyroid epithelial cells, generating an average of 40 visible colonies per 60-mm dish by 10 days (Figure 1A). Treatment with PMA, 3 days after retroviral infection, effectively abolished *ras*-induced colony formation (Figure 1C). In contrast, treatment with PMA together with GF 109203X resulted in the formation of a similar number of colonies to that in

**Figure 3** Effects of 5-day treatment with PMA and/or GF 109203X on cell growth and survival in semiconfluent cultures of R18 cells, with (filled columns) or without (open columns) induction of mutant *ras* expression by 75 μM $Zn^{2+}$

untreated cultures (Figure 1D), as did GF 109203X alone (Figure 1B) or DMSO carrier (not shown).

When this colony growth assay was repeated using the R18 thyroid cell line in place of primary cells, similar results were obtained. In untreated controls (with the inducible mutant *ras* gene switched off), approximately 350 colonies per 60-mm dish were obtained after 10 days. (R18 proliferation is driven by SV40 T and is not dependent on *ras*.) Treatment of such uninduced cells with PMA and/or GF 109203X had no effect on colony formation (Figure 2A–D). Cultures in which mutant *ras* expression was induced by the addition of 50 μM $Zn^{2+}$ (Figure 2E) showed a slight reduction in the number of colonies compared with uninduced controls (Figure 2A). Addition of PMA to cells expressing mutant *ras* resulted in a striking reduction in colony yield, with only a few small, ragged colonies surviving after 10 days (Figure 2G). However, simultaneous exposure to GF 109203X effectively blocked the toxic effect of PMA, resulting in an equivalent number of colonies to the untreated culture (Figure 2H). Again, addition of GF 109203X alone (Figure 2F) or the DMSO carrier (not shown) did not affect colony formation.

To demonstrate that the effects observed in the colony assays reflected not only growth inhibition but also actual cell loss, an analogous experiment was carried out using semiconfluent cultures of R18 cells. With mutant *ras* uninduced, treatment with PMA and/or GF 109203X had little effect on cell survival, cell number increasing by approximately 20-fold over an 8-day period irrespective of treatment. Induction of mutant *ras* by 75 μM $Zn^{2+}$ by itself caused a slight (1.3-fold) decrease in cell number (Figure 3). However, addition of PMA after *ras* induction resulted in massive cell death with a tenfold decrease in cell number after 8 days in comparison with uninduced control cultures. Phase-contrast microscopy revealed degenerative changes and cell detachment, leaving many refractile, blebbing cells floating free in the medium (Figure 4C). In contrast, simultaneous addition of GF 109203X greatly reduced the magnitude of cell death induced by PMA (Figures 3 and 4D), the cell number at 8 days not being significantly different from those in *ras*-expressing untreated cultures (Figure 3). As observed in the other assays, treatment with GF 109203X alone was without effect.

© Cancer Research Campaign 1998

**Figure 4** Phase-contrast micrographs of R18 cells, all expressing mutant *ras* induced by $Zn^{2+}$, 5 days after the following treatments: (**A**) untreated, (**B**) GF 109203X, (**C**) PMA and (**D**) PMA and GF 109203X

### *Effect of down-regulation of PKC*

R18 cells were plated at a clonal density (3 × $10^3$ cells per 60-mm dish). PMA (1 μg $ml^{-1}$) was added to one-third of the dishes immediately upon plating ('pretreatment' dishes – set C in Figure 5A), and the cells were incubated for a period of 3 days to allow down-regulation of PKC, as previously established in our system by measurement of phorbol ester binding sites (Bond et al, 1992). The remaining plates were left in normal medium ('treatment', set B, and 'control', set A). $Zn^{2+}$ (50 μM) was then added to one-half of each set of plates to induce the expression of mutant *ras*. After a further period of 2 days, 1 μg $ml^{-1}$ PMA was added to the 'treatment' plates, and all sets were assayed for the formation of colonies after 10 days.

In the absence of *ras* induction, similar numbers of colonies were found in all three conditions (Figure 5B a–c). With *ras* expressed (Figure 5B d–f), as before, PMA abolished colony formation (compare Figure 5B d and e). However, pretreatment with PMA greatly reduced the toxic effect of subsequent PMA treatment (compare Figure 5B e and f), although the 'protection' was less complete than that seen with GF 109203X.

### Role of specific PKC isoforms

### *PKC isoform expression*

An array of antibodies was used to investigate the expression and subcellular distribution of the conventional (α, βI, βII, γ), novel (δ, ε, η, θ) and atypical (ζ, τ and λ) PKC isoforms in the R18 cell line. Western analysis revealed expression of α and ε, together with ζ, ι and λ (Figure 6A), α and ε being localized predominantly in the cytoplasm, with a smaller proportion in the particulate fraction. Expression of β, γ, δ or θ was not detectable (not shown). As ζ, ι and λ are atypical isoforms and not affected by PMA, this indicates that the toxic effect of PMA is mediated by activation of one or both of the remaining isoforms α and ε.

### *Modulation of PKC α and ε by PMA*

The role of α and ε was examined further by analysing the subcellular redistribution of these isoforms after exposure of R18 cells to PMA.

In untreated cells, Western blot analysis showed PKC α to be predominantly cytosolic with a small amount detectable in the

© *Cancer Research Campaign 1998*

**Figure 5** Effect of PKC down-regulation on PMA-induced toxicity. (**A**) Experimental design of the R18 cell colony assay to test the effect of PKC down-regulation by pretreatment with PMA before induction of mutant *ras*. Experimental groups: set A, untreated controls; set B, 'standard' PMA treatment protocol; set C, PMA pretreatment. Hatched bars indicate periods of exposure to PMA. (**B**) R18 colony assay to test the effect of PKC down-regulation. Growth of colonies with mutant ras uninduced (a–c) and induced (d–f) 10 days after the treatments described in **A**

particulate fraction; PKC ε was more evenly distributed. Treatment with PMA (1 μg $ml^{-1}$) caused a rapid shift of both isoforms to the particulate and nuclear fractions, with little remaining detectable in the cytoplasmic fraction by 30 min (Figure 6B). (The faster running band in the blots of PKC α nuclear fraction in Figure 6A and B has subsequently been shown, using an alternative antibody, to be non-specific.)

Taken together, these results suggest that the critical biochemical event in the induction of cell death is translocation of PKC α and/or ε to membranes or nucleus.

### PMA stimulates MAPK translocation: a potential pro-apoptotic signal

The observation that PMA and mutant *ras* interact to bring about cell death could most readily be explained on the basis of 'cross-talk' between their respective downstream signal pathways. To begin to investigate this, we examined one candidate common pathway, the mitogen-activated protein kinase (MAPK) cascade, which is known to be stimulated by both PKC and ras in many cell types and which has been associated with both proliferogenic (Marshall, 1996) and, more recently (Kauffmann-Zeh et al, 1997), pro-apoptotic effects. MAPK activation in the R18 cell line was assessed indirectly by immunocytochemistry using an anti-p42 MAPK antibody to detect the cellular distribution of MAPK, cytoplasmic-to-nuclear translocation being a well-established correlate of MAPK activation (Traverse et al, 1992; Fukuda et al, 1997). In untreated cells (mutant *ras* uninduced) (Figure 7A) MAPK localization was predominantly cytoplasmic. Surprisingly, induction of mutant *ras* did not produce any detectable increase in the overall cellular content of MAPK or in its localization, as assessed after 2 days (Figure 7C) or earlier time-points (not shown). In contrast, PMA treatment caused a rapid redistribution of MAPK to the nucleus, very little remaining detectable in the cytoplasm after 30 min (Figure 7B). Importantly, a similar translocation of indistinguishable magnitude was produced by PMA in cells that were expressing mutant *ras* (Figure 7D).

© Cancer Research Campaign 1998

### PMA and mutant *ras* are synergistic in inducing apoptosis

To investigate in more detail the nature of the observed cell death, R18 cells were treated as usual with 75 μM $Zn^{2+}$ for 48 h to allow induction of mutant *ras* followed by PMA (1 μg $ml^{-1}$) and then analysed at multiple time-points for a further 120 h.

### *Electron microscopy*

Transmission electron microscopy of R18 cells expressing mutant *ras* 96 h after treatment with PMA revealed condensation of chromatin into dense patches in tight apposition to the nuclear envelope (Figure 8A), together with ruffling and blebbing of the plasma membrane.

### *FIGE analysis*

The above cultures also displayed high-molecular-weight (~50 kb) DNA fragmentation (Figure 8B) by FIGE analysis. As a positive control, these fragments were also observed (Figure 8B) in murine thymocytes treated with dexamethasone ($10^{-7}$ M) together with zinc acetate (1 mM), which has been shown to prevent the final internucleosomal cleavage stage of apoptosis (Cohen et al, 1992).

### *Terminal deoxynucleotidyl transferase (TdT) assay*

TdT assay was carried out both on attached cells and (when appropriate) on cytospin preparations of detached or loose cells.

Detached cells expressing mutant *ras* obtained from cultures treated for 72 or 96 h with PMA exhibited chromatin condensation and numerous TdT-labelled fluorescent bodies (Figure 8C). Interestingly, at earlier time-points (24 and 48 h), there was minimal detachment, with only a small proportion of these detached cells being TdT positive, indicating an unusually long lag period between the trigger for apoptosis and the onset of the 'execution' phase (Earnshaw, 1995). In contrast, the two positive controls (DOHH2 and C6 cell lines treated with etoposide) both showed strongly fluorescent, condensed chromatin and blebbing by 24 h (data not shown), and, beyond 48 h, showed complete degradation of DNA with TdT negativity. As expected, < 1% of untreated DOHH2 cells were positive.

Parallel analysis of attached R18 cells revealed very few TdT-positive cells, even after long periods of PMA treatment (data not shown), indicating that once the execution phase is initiated, cells rapidly detach. This was also observed in the attached population in the control cell line C6.

**Figure 6** Role of specific PKC isoforms. (**A**) Expression of PKC isoforms in R18 cells. Untreated cells were homogenized and separated into cytosolic (C), particulate (P) or nuclear (N) fractions and run on SDS–PAGE. Western analysis was performed using an array of specific antibodies against $\alpha$, $\beta$, $\gamma$, $\varepsilon$, $\delta$, $\theta$, $\iota$, $\lambda$ and $\zeta$ PKC isoforms revealing expression of $\alpha$ and $\varepsilon$ together with $\zeta$, $\iota$, $\lambda$. Expression of $\beta$, $\gamma$, $\delta$ and $\theta$ was not detectable. (**B**) Redistribution of PKC isoforms $\alpha$ and $\varepsilon$ in R18 cells upon 30 min treatment with PMA

### *Conventional gel electrophoresis*

Based on the TdT result, genomic DNA was extracted from loose cells expressing mutant *ras* after 96 and 120 h of PMA treatment and compared with DNA extracted from trypsinized untreated R18 cells, as a negative control. Conventional gel electrophoresis revealed internucleosomal cleavage with the production of classical apoptotic ladders at both time-points (Figure 8D).

Taken together, these four complementary techniques provide strong evidence to suggest that the mode of cell death induced by PMA in cells expressing mutant *ras* is apoptotic in nature.

A B C D

**Figure 7** Translocation of MAPK by PMA in R18 cells in the presence or absence of mutant ras expression. Subcellular distribution of MAPK shown by immunofluorescence using anti-p42 MAPK antibody in: (**A**) untreated R18 cells, (**B**) 30 min after addition of PMA, (**C**) cells expressing mutant ras for 2 days and (**D**) 30 min after PMA treatment of cells already expressing mutant ras

**Figure 8** Evidence that treatment of R18 cells expressing mutant *ras* with PMA induces apoptotic death. (**A**) Electron micrographs showing (a) untreated R18 cells, (b) R18 with mutant *ras* induced + PMA 96 h. Note dense patches of condensed chromatin associated with the nuclear envelope. (**B**) FIGE analysis of genomic DNA for high-molecular-weight chromatin cleavage. Lanes: (a) λ *Hin*dIII, (b) BioRad *S. cerevisiae* chromosomal DNA markers, (c) unrelated control cell line (Nθ-ts1) genomic DNA, (d) R18 with mutant *ras* induced + PMA 96 h, (e) untreated R18 cells, (f) murine thymocytes treated with dexamethasone and zinc acetate dihydrate and (g) untreated murine thymocytes. Note ~50 kb fragments characteristic of apoptosis in lanes (d) and (f). (**C**) Terminal deoxynucleotidyl transferase (TdT) assay of R18 cells expressing mutant *ras* + PMA 96 h. Chromatin condensation and fluorescent apoptotic bodies can be observed in a high proportion of these loose cells collected from the medium. (**D**) Conventional gel electrophoresis of genomic DNA for low-molecular-weight internucleosomal cleavage. (a) Untreated DOHH2 cells, (b) DOHH2 +24 h VP16 (0.25 μM), (c) untreated R18, (d) R18 with mutant *ras* induced +96 h PMA and (e) R18 with mutant *ras* induced +120 h PMA. DNA laddering can be observed at both 96 and 120 h after PMA treatment in R18 cells expressing mutant *ras*. A 1-kb ladder is shown for comparison

© Cancer Research Campaign 1998

### DISCUSSION

Our data confirm the ability of PMA to induce cell death in both primary and immortal thyroid cells in the presence, but not in the absence, of mutant *ras* expression. We have now shown that this cell death displays multiple characteristics of apoptosis, including high-molecular-weight DNA fragmentation (~50 kb fragments) and low-molecular-weight internucleosomal cleavage (~180–200 bp fragments). The latter, however, only becomes detectable approximately 4 days after exposure to PMA, an unexpectedly long delay that probably accounts for its having being overlooked in earlier work (Dawson et al, 1993). This delay suggests that a period of gene transcription is required, possibly involving the synthesis of essential components of the apoptotic pathway, such as fas or fas ligand. Such a phenomenon may also explain the apparently 'incomplete' apoptosis reported in another similar model, the response to PMA in MCF7 breast cancer cells over-expressing PKC α (de Vente et al, 1995).

On the assumption that conventional or novel PKC isoforms are the crucial pharmacological target of PMA in these experiments, there are two potential alternative mechanisms that might mediate PMA-induced toxicity: either initial stimulation or down-regulation of enzyme activity (Parker et al, 1995). We have shown here that an inhibitor of c and n PKCs, GF 109303X (Toullec et al, 1990), effectively abolishes the toxic effect of PMA in both primary cultures and the cell line model. This strongly suggests that it is the initial stimulation of PKC by PMA that induces toxicity, because, if this was a result of down-regulation, inhibition of PKC by GF 109203X would be expected to mimic, not inhibit, PMA-induced cell death. The finding that down-regulation of PKC by pretreatment with PMA is also protective provides further support for this conclusion and argues against the possibility that GF 109203X abrogates toxicity by a mechanism independent of PKC.

It should be noted that the failure of GF 109203X to prevent H-*ras*-induced mitogenesis in primary thyroid epithelial cells demonstrates that this response to *ras* is independent of conventional or novel PKC isoforms, although we cannot exclude the possible involvement of an atypical PKC, such as ζ, which has been shown to play a role in *ras*-induced mitogenic signalling in some other models, e.g. oocytes and NIH 3T3 fibroblasts (Berra et al, 1993).

Western blot analysis showed the presence of PKCs α, ε, ι, λ and ζ. As ι, λ and ζ are atypical isoforms and not affected by PMA, they can be eliminated as potential candidates, suggesting that α and/or ε are the critical isoforms for the induction of cell death. Consistent with this, Western analysis showed that a 30-min treatment with PMA induced translocation of PKC α and ε from the cytoplasm to the particulate and nuclear fractions. Further discrimination between α and ε was not attempted here but may be possible through use of isotype-specific constitutively active mutants (Schönwasser et al, 1998) or through differential down-regulation of one isoform by lower concentrations of PMA (Cai et al, 1997). Results from other models suggest that α and ε may function as alternative (redundant) signalling pathways (Cai et al, 1997).

The most obvious downstream target for PKC in this system is the MAPK pathway. PKC α (Kolch et al, 1993) and ε (Cai et al, 1997) have been shown to activate raf leading to subsequent activation of MAPK (Marquardt et al, 1994), although the exact mechanism (in particular the role of phosphorylation) is still disputed (Schönwasser et al, 1998). There is also evidence for raf-independent activation through alternative MEK kinases (Chao et al, 1994) or even at the level of MAPK itself (Grammer and Blenis, 1997). Furthermore, MAPK activation has been associated with apoptosis in several (although by no means all) cell models (Xia et al, 1995; Fukasawa and Vande Woude, 1997; Kauffmann-Zeh et al, 1997).

MAPK would also be expected to be activated by mutant *ras*, however, raising the possibility that PMA would not be able to produce any further increase in activity in mutant *ras*-expressing cells. The observation that PMA can still induce nuclear translocation of MAPK, a well-established (although admittedly indirect) index of MAPK activation (Fukuda et al, 1997), even in the presence of mutant *ras*, is therefore important in supporting the role of this pathway in PMA-mediated cell death.

The major mechanistic question raised by our data, however, is why thyroid cells are only killed by PMA in the presence of mutant *ras*. Although originally regarded as anti-apoptotic, it is now clear that *ras* activation in itself can generate anti- or pro-apoptotic effects, depending on the context (Lin et al, 1995; Fukasawa et al, 1997; Kauffmann-Zeh et al, 1997). In one well-characterized model (Kauffmann-Zeh et al, 1997), for example, use of *ras* effector mutants showed that the outcome can depend on the balance between *ras* signalling pathways, in this case PI3K being anti-apoptotic with MAPK and/or JNK being pro-apoptotic.

MAPK is an attractive candidate as it is the obvious point of convergence between *ras* and PMA, and we have previously postulated that the 'sensitizing' effect of mutant *ras* might operate by a simple additive interaction between *ras* and PMA on MAPK signalling, resulting in an excessive and therefore pro-apoptotic signal. Although this is not supported by the MAPK translocation data, which show no discernible difference between the MAPK response to PMA in the presence or absence of mutant *ras*, this cannot be regarded as conclusive, as translocation is an indirect index of activation, and it would be rather surprising if expression of mutant *ras* alone failed to result in any degree of sustained activation. It remains formally possible therefore that more direct analysis by assessment of MAPK phosphorylation status and in vitro kinase activity may still reveal such an additive interaction. Nevertheless, we clearly need to consider the alternative possibility that the sensitizing effect of *ras* operates via a different pathway, for which the MEKK/SAPKK/JNK cascade must currently be the strongest candidate (Xia et al, 1995; Johnson et al, 1996).

Distinction between these alternatives should be possible by direct manipulation of the MAPK or JNK pathways using constitutively active or dominant-negative mutants (Cowley et al, 1994), pharmacological inhibitors (Alessi et al, 1995; Cohen, 1997) or ras effector mutants (Kauffmann-Zeh et al, 1997). Whatever the outcome, the model that is emerging is that signals generated in the thyroid cell by mutant *ras* are in a state of delicate balance that is tipped in favour of apoptosis by the additional pro-apoptotic effect of PMA, mediated most probably via MAPK.

Experiments using a wide range of cell types have demonstrated either cytostatic or cytotoxic effects of supraphysiological *ras* signalling (Franza et al, 1986; Hirakawa and Ruley, 1988; Ridley et al, 1988), most recently in human fibroblasts (Serrano et al, 1997). In these models, however, cells were effectively rescued by loss of either the p53-and/or the pRb-mediated signal pathways. In contrast, in the human cell system described here, abrogation of both functions by SV40 T (which is expressed by both R18 and its parent line) does not appear to protect against PKC-induced cell death in the presence of mutant *ras*. This is potentially of therapeutic significance, as it suggests that PKC stimulators would be effective even against tumours that had lost p53 function.

Given the high frequency of *ras* mutations in many tumour types, the specificity of PMA-induced toxicity for thyroid epithelial cells expressing mutant *ras* increases the interest in PKC as an intracellular target for therapy.

On a purely empirical basis, one potent PKC activator, bryostatin 1, has already been shown to exert cytostatic or cytotoxic effects against some types of cancer cells both in vitro and in vivo (Gescher, 1992; Hornung et al, 1993) and has shown sufficient promise to be taken into clinical trials for treatment of melanoma and leukaemia (Philips et al, 1993; Prendiville et al, 1993). Unfortunately, however, in our model, bryostatin appears to be much less effective than PMA at all doses tested (Dawson et al, 1993).

One major biological property of PMA, not shared by bryostatin, is the ability to promote mutagen-induced experimental skin tumours (Hennings et al, 1987), related probably to its ability to induce keratinocyte differentiation (Szallasi et al, 1994). This raises the possibility that the cytotoxic effect of PMA against mutant *ras*-expressing cells might be inextricably linked to its tumour-promoting activity. Nevertheless, the pharmacological difference between bryostatin and PMA that most closely correlates with their different tumour-promoting properties is the inability of bryostatin to down-regulate PKC-δ (Szallasi et al, 1994). This difference is consistent with the suspected importance of chronic down-regulation rather than acute activation of PKC in tumour promotion by phorbol esters (Blumberg, 1991; Droms and Malkinson, 1991). Our model, however, depends solely on stimulation of PKC isoforms, and the lack of potency of bryostatin compared with PMA here is therefore more likely to be related to differences in its ability to stimulate PKC, such as the slower kinetics of PKC α and ε stimulation observed in keratinocytes (Szallasi et al, 1994). This is important for future work, as it supports the feasibility of designing an agent that would be as effective as PMA in killing cells expressing mutant *ras* but without its tumour-promoting properties.

Even if such an ideal agent was unavailable, however, the therapeutic potential of phorbol esters should not be written off simply on the historical basis of experimental skin tumour promotion. As mentioned above, there is much evidence to suggest that this results from long-term down-regulation of PKC (Blumberg, 1991; Droms and Malkinson, 1991), whereas the potential tumoricidal action reported here is an acute response dependent on short-term stimulation of PKC and should therefore require only 'bolus' administration. There is no evidence that such use would lead to increased risk of second tumours, and our preliminary toxicological studies in rats have shown no obvious signs of acute toxicity after systemic administration of phorbol esters.

Therapeutic use of such agents may therefore be a much more realistic option than might appear at first sight. Although the range of susceptible epithelial cell types has not yet been explored, an obvious target would be pancreatic cancer, which has a very high ras mutation rate and which currently carries a dismal prognosis.

### ACKNOWLEDGEMENTS

We thank Dr Gerald Cohen (Leicester) for his constructive advice on FIGE analysis of genomic DNA and Professor Paul Smith (Cardiff) for his help in the analysis of cell death by TdT. C6 and DoHH2 cells were kindly supplied by Dr J Gannon, ICRF, London, and Dr JC Kluin-Nelemans, Leiden, The Netherlands, respectively. This work was supported by the UK Cancer Research Campaign and the Medical Research Council.

### REFERENCES

Alessi DR, Cuenda A, Cohen P, Dudley DT and Saltiel AR (1995) PD-098059 is a specific inhibitor of the activation of mitogen activated protein kinase kinase in vitro and in vivo. *J Biol Chem* **270**: 27489–27494

Almoguera C, Shibata D, Forrester K, Martin J, Arnheim N and Perucho M (1988) Most human carcinomas of the exocrine pancreas contain mutant c-k-*ras* genes. *Cell* **53**: 549–554

Berra E, Diaz-Meco MT, Dominguez I, Municio MM, Sanz L, Lozano J, Chapkin RS and Moscat J (1993) Protein kinase C ζ isoform is critical for mitogenic signal transduction. *Cell* **74**: 555–563.

Blumberg PM (1991) Complexities of the protein kinase C pathway. *Mol Carcinogen* **4**: 339–344

Bond J, Dawson T, Lemoine N and Wynford-Thomas D (1992) Effect of serum growth factors and phorbol ester on growth and survival of human thyroid epithelial cells expressing mutant *ras*. *Mol Carcinogen* **5**: 129–135

Bos JL, Fearon ER, Hamilton SR, Verlaan-de Vries M, van Boon JH, van der Eb AJ and Vogelstein B (1987) Prevalence of *ras* gene mutations in human colorectal cancers. *Nature* **327**: 293–297

Cai H, Smola U, Wixler V, Eisenmann-Tape I, Diaz-Meco MT, Moscat J, Rapp and Cooper GM (1997) Role of diacylglycerol-regulated protein kinase C isotype in growth factor activation of the raf-1 protein kinase. *Mol Cell Biol* **17**: 732–741

Chao T-S O, Foster DA, Rapp UR and Rosner MR (1994) Differential raf requirement for activation of mitogen-activated protein kinase by growth factors, phorbol esters and calcium. *J Biol Chem* **269**: 7337–7341

Cohen GM, Sun X, Snowden RT, Dinsdale D and Skilleter DN (1992) Key morphological features of apoptosis may occur in the absence of internucleosomal DNA fragmentation. *Biochem J* **286**: 331–334

Cohen P (1997) The search for physiological substrates of MAP and SAP kinases in mammalian cells. *Trends Cell Biol* **7**: 353–361

Compere SL, Baldacci PA, Sharpe AH and Jaenisch R (1989) Retroviral transduction of the human c-Ha-ras-1 oncogene into mid-gestation in mouse embryos promotes rapid epithelial hyperplasia. *Mol Cell Biol* **9**: 6–14

Cowley S, Paterson H, Kemp P and Marshall CJ (1994) Activation of MAP kinase is necessary and sufficient for PC 12 differentiation and transformation of NIH 3T3 cells. *Cell* **77**: 841–852

Dawson T, Bond J, Eccles N and Wynford-Thomas D (1993) Toxicity of phorbol esters for human epithelial cells expressing a mutant *ras* oncogene. *Mol Carcinogen* **8**: 280–289

de Vente JE, Kukoly CA, Bryant WO, Posekany KJ, Chen J, Fletcher DJ, Parker PJ, Pettit GJ, Lozano G, Cook PP and Ways DK (1995) Phorbol esters induce death in MCF-7 breast cancer cells with altered expression of protein kinase C isoforms. *J Clin Invest* **96**: 1874–1886

Dekker LV and Parker PJ (1994) Protein kinase C – a question of specificity. *Trends Biochem Sci* **19**: 73–77

Droms KA and Malkinson AM (1991) Phorbol ester-induced tumour promotion by downregulation of protein kinase C. *Mol Carcinogen* **4**: 1–2

Earnshaw WC (1995) Apoptosis: lessons from in vitro systems. *Trends Cell Biol* **5**: 217–220

Franza BR, Maruyama K, Garrels JI and Ruley HE (1986) In vitro establishment is not a sufficient prerequisite for transformation by activated *Ras* oncogenes. *Cell* **44**: 409–418

Fukasawa K and Vande Woude GF (1997) Synergy between the Mos/mitogen-activated protein kinase pathway and loss of p53 function in transformation and chromosome instability. *Mol Cell Biol* **17**: 506–518

Fukuda M, Gotoh Y and Nishida E (1997) Interaction of MAP kinase with MAP kinase kinase: its possible role in the control of nucleocytoplasmic transport of MAP kinase. *EMBO J* **15**: 1901–1908

Gescher A (1992) Towards selective pharmacological modulation of protein kinase C – opportunities for the development of novel antineoplastic agents. *Br J Cancer* **66**: 10–19

Grammer TC and Blenis J (1997) Evidence for MEK-independent pathways regulating the prolonged activation of the ERK-MAP kinases. *Oncogene* **14**: 1635–1642

Greif H, Ben-Chaim J, Shimon T, Bechor E, Eldar H and Livine E (1992) The protein kinase C related PKC-L (η) gene product is localized in the cell nucleus. *Mol Cell Biochem* **12**: 1304–1311

Hennings H, Blumberg PM, Pettit GR, Herald CL, Shores R and Yuspa SH (1987) Bryostatin 1, an activator of protein kinase C, inhibits tumour promotion by phorbol esters in SENCAR mouse skin. *Carcinogenesis* **8**: 1343–1346

Hirakawa T and Ruley HE (1988) Rescue of cells from *ras* oncogene-induced growth by arrest by a second, complementing, oncogene. *Proc Natl Acad Sci USA* **85**: 1519–1523

© Cancer Research Campaign 1998

Hornung RL. Pearson JW. Beckwith M and Longo DI (1993) Preclinical evaluation of bryostatin as an anticancer agent against several murine tumour cell lines: in vitro versus in vivo activity. *Cancer Res* **52**: 101–107

Johnson NL. Gardner AM. Diener KM. Langecarter CA. Gleavy J. Jarpe MB. Minden A. Karin M. Zon LI and Johnson GL (1996) Signal transduction pathways regulated by mitogen-activated extracellular response kinase kinase kinase induce cell-death. *J Biol Chem* **271**: 3229–3237

Kauffmann-Zeh A. Rodriguez-Viciana P. Ulrich E. Gilbert C. Coffer P. Downward J and Evan G (1997) Suppression of c-Myc-induced apoptosis by Ras signalling through PI(3)K and PKB. *Nature* **385**: 544–548

Kolch W. Heidecker G. Kochs G. Hummel R. Vahidi H. Mischak H. Finkenzeller G. Marme D and Rapp UR (1993) Protein kinase C α activates RAF-1 by direct phosphorylation. *Nature* **364**: 249–252

Lemoine NR. Mayall ES. Wyllie FS. Williams D. Goyns M. Stringer B and Wynford-Thomas D (1989) High frequency of *ras* oncogene activation in all stages of human thyroid tumorigenesis. *Oncogene* **4**: 159–164

Lin H-J. Eviner V. Prendergast GC and White E (1995) Activated H-ras rescues E1A-induced apoptosis and co-operates with E1A to overcome p53-dependent growth arrest. *Mol Cell Biol* **15**: 4536–4544

Malcomson RDG. Oren M. Wyllie AH and Harrison DJ (1995) p53-independent death and p53-induced protection against apoptosis in fibroblasts treated with chemotherapeutic drugs. *Br J Cancer* **72**: 952–957

Marquardt B. Frith D and Stabel S (1994) Signalling from TPA to MAP kinase requires protein kinase C. raf and MEK: reconstitution of the signalling pathway *in vitro*. *Oncogene* **9**: 3213–3218

Marshall CJ (1996) Ras effectors. *Current Biol* **8**: 197–204

Namba H. Rubin SA and Fagin JA (1990) Point mutations of *ras* oncogenes are an early event in thyroid tumorigenesis. *Mol Endocrinol* **4**: 1474–1479

Ono Y. Fujii T. Ogita KT. Kikkawa K. Igarashi K and Nishizuka Y (1988) Protein kinase C ζ subspecies from rat brain: its structure expression and properties. *Proc Natl Acad Sci USA* **86**: 3099–3103

Parker PJ. Bosca L. Dekker L. Goode NT. Hajibagheri N and Hansra G (1995) Protein kinase C (PKC)-induced PKC degradation: a model for down-regulation. *Biochem Soc Trans* **23**: 53–55

Philips PA. Rea D. Thavasu P. Carmichael J. Stuart NSA. Rockett H. Talbot DC. Ganesan T. Pettit GR. Balkwill F and Harris AL (1993) Phase 1 study of bryostatin 1: assessment of interleukin 6 and tumor necrosis factor α induction *in vivo*. *J Natl Cancer Inst* **85**: 1812–1818

Prendiville J. Crowther D. Thatcher N. Woll PJ. Fox BW. McGown A. Testa N. Stern P. McDermott R. Potter M and Pettit GR (1993) A phase 1 study of bryostatin 1 in patients with advanced cancer. *Br J Cancer* **68**: 414–424

Ridley AJ. Paterson HF. Noble M and Land H (1988) *ras*-mediated cell cycle arrest is altered by nuclear oncogenes to induce Schwann cell transformation. *EMBO J* **7**: 1635–1645

Sambrook J. Fritsch EF and Maniatis T (1989) *Molecular Cloning: A laboratory manual* 2nd edn. New York: Cold Spring Harbor Laboratory Press

Schonwasser DC. Marais RM. Marshall CJ and Parker PJ (1998) Activation of the mitogen-activated protein kinase/extracellular signal-regulated kinase pathway by conventional. novel and atypical protein kinase C isotypes. *Mol Cell Biol* **18**: 790–798

Serrano M. Lin AW. McCurrach ME. Beach D and Lowe SW (1997) Oncogenic ras provokes premature cell senescence associated with accumulation of p53 and $p16^{ink4a}$. *Cell* **88**: 593–602

Stanwell C. Gescher A. Bradshaw TD and Pettit GR (1994) The role of protein kinase C isoenzymes in the growth and inhibition caused by bryostatin 1 in human A549 lung and MCF-7 breast carcinoma cells. *Int J Cancer* **56**: 585–592

Suarez HG. du Villard JA. Severino M. Caillou B. Schlumberger M. Tubiana M. Parmentier C and Monier R (1990) Presence of mutations of all three *ras* genes in human thyroid tumors. *Oncogene* **5**: 565–570

Szallasi Z. Denning MF. Smith CB. Dlugosz AA. Yuspa SH. Pettit GR and Blumberg PM (1994) Bryostatin 1 protects protein kinase C-δ from down-regulation in mouse keratinocytes in parallel with its inhibition of phorbol ester-induced differentiation. *Mol Pharmacol* **46**: 840–850

Toullec D. Pianetti P. Coste H. Bellevergue P. Grand-Perret T. Ajakane M. Bandet V. Boissins P. Boursier E. Loriolle F. Duhamel L. Charons D and Kirilovsky J (1990) The bisindolylmaleimide GF 109203X is a potent and selective inhibitor of protein kinase C. *J Biol Chem* **266**: 15771–15781

Traverse S. Gomez N. Paterson H. Marshall C and Cohen P (1992) Sustained activation of the mitogen-activated protein (MAP) kinase cascade may be required for differentiation of PC12 cells. *Biochem J* **288**: 351–355

Wang C. Constantinescu SN. MacEwan DJ. Strulovici B. Dekker LV. Parker PJ and Pfeffer LM (1993) Interferon α induces protein kinase C-ε (PKC-ε) gene expression and a 4.7-kb PKC ε related transcript. *Proc Natl Acad Sci USA* **90**: 6944–6948

Williams DW. Wynford-Thomas D and Williams ED (1987) Control of human thyroid follicular cell proliferation in suspension and monolayer culture. *Mol Cell Endocrinol* **51**: 33–40

Xia Z. Dickens M. Raingeaud J. Davis RJ and Greenberg ME (1995) Opposing effects of ERK and JNK-p38 MAP kinases on apoptosis. *Science* **270**: 1326–1331

© Cancer Research Campaign 1998